# Long-term Outcomes of Childhood Onset Nephrotic Syndrome

**DOI:** 10.3389/fped.2016.00053

**Published:** 2016-05-25

**Authors:** Rebecca Hjorten, Zohra Anwar, Kimberly Jean Reidy

**Affiliations:** ^1^Pediatrics Nephrology, Children’s Hospital at Montefiore, Albert Einstein College of Medicine, Bronx, NY, USA

**Keywords:** nephrotic syndrome, children, pediatric, outcomes, genetics, focal glomerulosclerosis, minimal-change disease

## Abstract

There are limited studies on long-term outcomes of childhood onset nephrotic syndrome (NS). A majority of children with NS have steroid-sensitive nephrotic syndrome (SSNS). Steroid-resistant nephrotic syndrome (SRNS) is associated with a high risk of developing end-stage renal disease. Biomarkers and analysis of genetic mutations may provide new information for prognosis in SRNS. Frequently relapsing and steroid-dependent NS is associated with long-term complications, including dyslipidemia, cataracts, osteoporosis and fractures, obesity, impaired growth, and infertility. Long-term complications of SSNS are likely to be under-recognized. There remain many gaps in our knowledge of long-term outcomes of childhood NS, and further study is indicated.

## Introduction

Nephrotic syndrome (NS) is characterized by proteinuria, edema, hypoalbuminemia, and hyperlipidemia. The seminal studies of childhood onset NS were performed in the 1970s, in the International Study of Kidney Disease in Children (ISKDC) (1, 2). The ISKDC studies established the most likely pathologic diagnoses of childhood NS, as biopsies were performed at presentation in all 127 patients. Overall, the most common diagnosis (*n* = 95/127) was minimal-change disease (MCD), which was present in 94% of the children with steroid-sensitive nephrotic syndrome (SSNS) (1). In steroid-resistant nephritic syndrome (SRNS), focal glomerulosclerosis (FSGS) was the most common histopathologic lesion (1). The ISKDC established that the majority of children with NS (80%) respond to corticosteroid treatment (1, 2). Thus, the ISKDC established the paradigm of treating all children presenting with NS with corticosteroids and only performing biopsies in steroid resistant patients (2).

Since the ISKDC, the majority of studies looking at outcomes classified patients by response to corticosteroids or other therapy and by NS relapse. The least severe course with SSNS is associated with few or no relapses. More complicated are those with SSNS with frequent relapses (FR = >2 relapses over a 6-month or >3 over a 12-month period) or steroid dependence (SD = relapses during treatment or within 2 weeks of stopping corticosteroids) (3). Some patients with initial steroid response develop SRNS (late non-responders) and others present with SRNS. Finally, there are the most severe cases of NS that are both corticosteroid and other treatment resistant. Long-term renal-related complications of childhood NS would include NS relapse in adulthood, hypertension, chronic kidney disease (CKD), and end-stage renal disease (ESRD). Long-term non-renal complications include complications of corticosteroids and other immunosuppressant medications, including effects on growth, bone health, fertility, and risk for malignancy. An additional consideration are psychosocial issues associated with NS. As with many chronic diseases, NS can represent a barrier to attaining educational degrees and employment and/or developing stable relationships/marriage. Indeed, a quality-of-life (QOL) survey of children with NS in the National Institutes of Health Focal Segmental Glomerulosclerosis (NIH FSGS) clinical trial revealed that children with SRNS had poor QOL scores similar to children on dialysis (4).

Here, we will review the current data on long-term NS outcomes, potential new predictors of treatment and renal outcomes, and gaps in our knowledge that warrant further investigation.

## Outcomes in SRNS

It is clear that the worst outcomes occur in SRNS, with 34–64% progressing to ESRD within 10 years of diagnosis (5–8). The majority of SRNS patients are treated with second-line agents, such as calcineurin inhibitors and other immunosuppressant medications. There is a highly variable response, which may depend upon the population studied. For example, studies of calcineurin inhibitors report divergent response rates of between 25 and 75% in children with SRNS (9). This variability in response to calcineurin inhibitors likely reflects in part differences dependent on the underlying histopathology of the SRNS, with MCD more likely to respond than FSGS. Other factors, such as race or ethnicity of the population being studied, may also affect likelihood of response (10). In particular, African-American and Hispanic children with FSGS have poorer outcomes, with one study demonstrating 50% progress to ESRD within 3 years (11, 12). What is clear is that response to any therapy is a major prognostic factor (13). Abeyagunawardena et al. examined 10-year outcomes of 66 children with SRNS. Response to therapy was associated with 90% renal survival, while almost 50% with failure to respond to therapy had progressed to ESRD (13). Response to second-line immunosuppressant therapy and risk of progression may differ in children who initially respond to therapy, but then become non-responsive (late non-responders). Straatmann et al. demonstrated that up to 87% of late non-responders in a multicenter study initially responded to a calcineurin inhibitor (14). However, long term, 31% of the cohort became non-responsive, and had decreased renal function and increased progression to ESRD compared to those that remained treatment responsive (14).

## Biomarkers to Predict Response to Therapy

One of the largest areas of research has been the search for soluble factors that may be predictive of treatment response and may also serve as targets for future therapies. This interest was partly spurred due to studies showing that serum from patients with FSGS can induce proteinuria in rats and increase the glomerular permeability of isolated glomeruli (15). In addition, plasmapheresis has been able to induce remission in some patients with recurrent FSGS post transplant (16, 17).

One circulating factor that has been studied is the soluble urokinase plasminogen activator (suPAR). Wei et al. showed in a study that 94 children with FSGS and 70 adults and children with FSGS had elevated levels of suPAR when compared with healthy controls (18). Peng et al. then showed, in a group of 176 children with idiopathic NS followed for approximately 30 months, that suPAR levels were able to predict steroid resistance (19). However, these results have not been widely reproducible, questioning the role of suPAR to distinguish FSGS from other glomerular diseases (20–22). For example, Sinha et al. in a cross-sectional cohort study of 83 healthy pediatric controls versus 469 patients with NS, 138 of which had SSNS, was not able to show an association of suPAR levels with either SSNS versus SRNS. In the same study, suPAR levels were not significantly associated with the diagnosis of FSGS (22). This study demonstrated that suPAR had an inverse correlation with glomerular filtration rate, and some have proposed it may be a predictor of renal progression (22, 23).

Other investigators have looked to evidence of immune activation to predict response to immunosuppressive treatment. One study of 26 children with idiopathic SSNS that were followed for less than a year showed a differential pattern of regulatory Th1 and Th2 cells in patients in remission versus in relapse (24). Another study of 46 children with NS demonstrated that T-cell glucocorticoid receptor expression was higher in patients with NS responsive to steroid therapy and in patients with MCD, when compared to patients with FSGS (25). In one study of 17 patients with MCD and 22 with FSGS, urinary CD80 (present on antigen-presenting cells and podocytes) was able to distinguish MCD from FSGS. Another investigator determined that the urinary CD80 level associated with disease remission (26, 27). In a search for novel, yet unrecognized, markers of both steroid response and disease progression, investigators have used unbiased approaches to examine gene expression, the serum and urine proteome, and the metabolome (28–30). One group took serum from blood of 33 children with idiopathic SSNS at presentation, in remission while on medication, and in remission once medication was stopped. Evaluating serum peptide levels at all time points, they identified a novel marker, apolipoprotein AII. The levels of apolipoprotein AII correlated with degree of proteinuria at presentation and decreased with remission (31).

All these studies are limited by small numbers of patients and most are single-center studies. Poor reproducibility has plagued efforts to identify biomarkers in NS (32, 33). Thus, newly available large cohorts of NS patients, such as Podonet (34), NEPTUNE (35), and Cure GN that include well-characterized pediatric patient populations of NS are critical for discovery and validation of new biomarkers.

## Genetic Factors in Predicting Outcome in NS

One of the new factors that may predict response to therapy and renal outcomes are genetic variants of NS. Genetic mutations are most likely to be identified in congenital nephrotic syndrome (CNS). Mutations in NPHS1, NPHS2, LAMB2, and WT-1 were identified in two-thirds of a largely European cohort of 89 infants with NS under the age of 1 (36). The overall average age of ESRD was 5.6 years. While the numbers were small and many outcomes were not known, patients with NPHS1 mutations had ESRD at an average age of 4.6 years, whereas those with NPHS2 mutations had ESRD at an average age of 7.4 years (36). It is possible these differences could be explained by differences in clinical course and management of congenital NS (e.g., nephrectomies), rather than the genetic basis of disease. However, it suggests that additional studies should address whether specific genetic mutations correlate with outcomes in early onset NS.

There are well over 40 genetic mutations associated with FSGS, and new mutations continue to be identified (37). A single-gene mutation may be identified in up to 29% of patients with SRNS onset prior to age 25 (38). Patients with genetic mutations are less likely to respond to immunosuppressant therapy and more likely to develop ESRD (39). One of the largest studies by Buscher et al. examined renal outcomes of at 10-year follow-up of 231 children in a European cohort with SRNS. For those presenting after 3 months of age, 58% children with SRNS associated with genetic mutations had progressed to ESRD, versus 29% with SRNS and no genetic mutation identified (39). Recently, genetic variants in *APOL1* were identified as risk factors for renal disease in people of African descent. Carrying two copies of *APOL1* coding variants (G1 and G2) is a risk factor for hypertensive nephropathy, lupus nephropathy, FSGS, and HIV nephropathy (40). Recent analysis of the FSGS Clinical Trial, a NIH supported randomized controlled trial comparing MMF to cyclosporine in children and young adults (41), demonstrated that 67–72% of 94 patients of African descent in the study harbored two copies of the *APOL1* risk variants, and they were more likely to progress to ESRD, with 40% reaching ESRD within approximately 8 years (42). Of note, APOL1 risk variants did not associate with response to therapy, although a small percentage responded to either therapy (41). Many genetic analyses, to date, have focused on largely European and homogeneous populations; the cohorts of NEPTUNE (35, 43) and CureGN provide an opportunity to understand the contribution of gene–gene and gene-environmental interactions in modulating long-term outcomes.

Recently, two genes have been identified associated with treatment sensitive NS. Epithelial membrane protein 2 (EMP2) was identified in familial SSNS (44). While only a few patients were identified, the majority had no renal failure after over 20 years of follow-up (44). Overall, one can be optimistic that, in the future, identification of specific genetic mutations may help guide therapy and provide prognostic information to families.

## Long-Term Complications of Steroid-Sensitive Nephrotic Syndrome are Likely Under-Recognized

There is increasing focus on a life-course approach to optimizing health outcomes, with recognition that exposures in childhood establish the risk for adult-onset disease (45). The vast majority of children will have SSNS and MCD, which is thought to have benign long-term outcomes. However, recent studies have suggested that other conditions that were thought to be benign, such as congenital solitary kidneys, are associated in increased risk of hypertension and ESRD in adulthood (46). The increased risk of ESRD required more than 30 years to manifest, and thus was unlikely to be detected without a concerted effort to study outcomes in these patients (46). Potential long-term complications of SSNS are likely to be under-recognized, as patients are often lost to follow-up.

## Outcomes in SSNS

There are a handful of studies looking at long-term outcomes of childhood onset SSNS (Table 1). All studies include either exclusively or mostly FR/SD NS patients.

**Table 1:** **Long-term complications of childhood SSNS**.

	Complication	Reported prevalence	Comment
Renal	Relapses in adulthood	5–40% (47, 48)	Higher risk with early onset, more frequent relapses in childhood
	Decreased GFR/ESRD	<1% at 20-year follow-up (48)	
	Hypertension	6–46% (49, 50)	Highest in FRNS with adult relapses
Immunosuppression-related	Overweight/obesity	8–23% (50, 51)	
	Growth failure	8–16% (48, 50)	
	Osteoporosis	13–63% (49, 50)	Highest in FRNS with adult relapses
	Fractures	20% (51)	
	Cataracts	6–20% (49, 50)	Highest in FRNS with adult relapses
	Infertility	Up to 75–94% (49, 51)	Highest with cytotoxic therapy
	Malignancy	Unknown	
Psychosocial	Educational status and employment	<10% failure to complete high school and <45% unemployed (51)	Single-center study
	Marital status/stable relationships	<40% unmarried or not in stable relationship (51)	Single-center study

## Risk of Relapse as an Adult

An early study by Trompeter et al. suggested that only 5% of children will have persistent relapses as adults (47). However, several recent studies suggest that FRNS is common, and relapses may persist into adulthood. Esfahani et al. examined the course of NS of over 200 children from Iran with follow-up between 5 and 20 years (52). Eighty-three percent experienced relapses in childhood and over 50% required additional immunosuppressive treatment, likely indicating FR/SD NS. Less than half the cohort sustained a remission for more than 3 years. Similarly, Ishikura et al. performed 10-year follow-up of a randomized controlled trial of cyclosporine for FR/SDNS in Japan. Fifty percent continued to relapse and remain on immunosuppressives in adulthood (50).

Fakhouri et al. studied 102/117 children admitted with SSNS in France using mailed questionnaires or phone calls to patients and/or their parents or attending physicians (48). More than 40% of the patients relapsed in adulthood (48). Younger age of NS onset, second-line immunosuppressant therapy, and more severe disease in childhood were associated with increased risk of relapse in adulthood. However, relapse rate in the first 6 months of disease was not predictive of adult relapses. Kabuki et al. also examined age of presentation effects on risk of relapse in 60 patients diagnosed with NS before age 10 in Japan with over 10 years follow-up (53). The majority (51/60) patients were relapse-free over 3 years. Early presentation (between age 1 and 3.9 years) was associated with more relapses and a longer interval between onset of NS and long-term remission. Conversely, older onset (after age 7 years) was associated with less active disease at 10 years follow-up.

Skrzypczyk et al. examined outcomes of 61/118 children with NS in Poland using a written questionnaire (51). Over 90% were SSNS and only 30% FRNS. However, 77% required second-line immunosuppressive therapy in addition to steroids. The patients had particularly benign courses in that all patients achieved remission and had normal renal function at the age of 18. Still 16% relapsed in adulthood, and risk of relapse as adult was associated with relapses as a child.

One of the longest follow-up studies was by Ruth et al. on 42 children with NS from Switzerland with a median follow-up of 22 years. Thirty-three percent of patients had relapses as adults. Number of relapses during childhood and adolescence and requirement for second-line therapy were risks for relapses in adulthood (54).

## Renal and Non-Renal Complications

Several of these studies examined long-term complications of immunosuppression. Ishikura et al. identified that on 10-year follow-up of exclusively FR/SDNS patients, a substantial number had short stature (13%), osteoporosis (13%), obesity (8%), cataracts (6%), and hypertension (6%) (50). Fakhouri et al. obtained information in their SSNS patients with 40% rate of relapse in adulthood (48). Of them, 7% had hypertension, and 1 out of the 102 patients had developed ESRD. The most common complications were likely long-term side effects of corticosteroids: 16% had short stature, 20% were overweight or obese, and 63% had osteoporosis.

One of the few direct assessments of long-term outcomes was performed by Kyrieleis et al. in 15 adult patients from the Netherlands with history of childhood onset FRNS who were still relapsing as adults (mean follow-up 24 years). Forty-six percent of patients had hypertension. Dual-energy X-ray absorptiometry identified osteoporosis in one-third of patients. Ophthalmologic examination identified cataracts in 3/15 (20%) of the patients. Six out of eight (75%) had abnormal semen examination with oligozoospermia, reduced sperm motility, and teratozoospermia.

The study of 61 patients with childhood NS from Poland by Skrzypczyk et al. demonstrated a substantial number of immunosuppressant-related complications. Sixteen percent were hypertensive, 23% were overweight, and 4.9% obese. Almost 20% had bone fractures and 8% had short stature (height <third percentile). Childlessness was more common (94%) in those treated with cytotoxic agents versus those not treated with cytotoxic agents. No increased risk of malignancy, infection, or cardiovascular events was detected.

Skrzypczyk et al. was one of the only studies to examine long-term psychosocial impact of NS as a chronic disease (51). In their follow-up study, 60.7% patients were in a steady relationship/married, 40.9% pursued higher education, and only 6.6% did not complete a high school (secondary) education. At the time of the study, 55.7% patients reported active employment.

Of note, none of studies that examined GFR observed a change (49, 51). The study with the longest follow-up (22 years) by Ruth et al. identified no effects on growth, obesity, or GFR, but did identify an effect on fertility (54). Only 8/42 patients had children, and cytotoxic therapy was associated with childlessness (54).

Nephrotic syndrome leads to endothelial dysfunction in children (55) and engenders a risk for artherosclerosis in adults (56, 57). In one study of 30 patients with childhood onset SSNS, dysregulation of lipids, including increased total cholesterol, low-density lipoprotein cholesterol, and homocysteine were persistent on follow-up at 4–15 years after completion of steroids (57).

## Gaps in Our Knowledge of Long-Term Outcomes of SSNS

The majority of the above studies are retrospective chart reviews or utilized surveys, with the inherent biases of those types of study design. Virtually, all studies lack a control population, which is relevant for outcomes, such as obesity and hypertension, which may be common in the adult population. Often they represent a single-center experience and have small numbers. Even the largest studies report outcomes from countries with homogenous populations, with limitations in their generalizability to ethnically diverse populations. Finally, the average length of follow-up in most studies was approximately 10 years, well short of the 30-year follow-up required to identify effects of a solitary kidney on ESRD outcomes (46).

It seems possible that, as with other conditions previously thought to be entirely benign, SSNS may engender increased risk of hypertension, atherosclerosis, CKD, and ESRD in adulthood (Table 2). In addition, there are insufficient studies of patient-reported outcomes, including QOL assessments. There are limited data on implications of childhood NS on educational attainment, employment, and marital status. Better understanding of potential adverse consequences may allow for interventions targeting improvement of both patient-reported and psychosocial outcomes. Our lack of knowledge limits effective transition of children with NS to adult providers. Those with few or no relapses are likely not to follow-up or to follow-up rarely with a pediatric nephrologist as they approach adulthood. An adult nephrologist is unlikely to follow these patients, as the majority will not have renal impairment at entry into adulthood. So the major question becomes how and when to communicate the potential for long-term issues with adult providers. One study in Japan demonstrated that the majority of practitioners had no transition plan in place for children with SSNS (58).

**Table 2:** **Gaps in knowledge**.

Gaps in knowledge	Opportunities to address
**Life-course effects of childhood NS**	Registries of childhood onset nephrotic syndrome
Are there risks of SSNS after 30 years? Are patients with childhood onset NS at higher risk of hypertension, CKD, and ESRD?	cNEPTUNE (pediatric cohort of incident nephrotic syndrome patients)
**Genetic factors and biomarkers**	Multicenter collaborative cohorts of pediatric onset NS
Can permeability factors or other biomarkers enable personalized approach to treatment?	
Can screening for genetic mutations or APOL1 variants in childhood onset NS stratify those children at highest risk for hypertension and renal disease in adulthood?	
**Psychosocial impact and patient-reported outcomes**	Multicenter collaborative cohorts of pediatric onset NS
Quality of life is significantly affected in childhood onset NS, particularly with severe disease. What is the effect on patient-reported outcomes? What is the long-term effect on educational status, employment and marital/stable relationships and are there interventions to improve outcomes?	
**Transition into adulthood**	Registries and multicenter collaborative cohorts of pediatric onset NS
(1) When should children with NS receive counseling about potential risks in adulthood?	
(2) How will adult providers be made aware of potential for complications in adults with a history of childhood NS?	
(3) Are screenings for artherosclerosis, osteoporosis, cataracts, infertility, and/or malignancy indicated?	
(4) What is the optimal transition plan for children with SSNS as they enter adulthood?	

Patient registries and follow-up of pediatric cohorts, such as cNEPTUNE (incident children with NS), may help provide answers to these questions.

## Conclusion

Underlying renal pathology, genetic factors, and ethnicity likely modulate response to treatment and progression of ESKD. Well-characterized and prospectively followed cohorts provide an exciting opportunity to improve our understanding of the ability of biomarkers and genetics to predict outcomes. Many of the long-term complications of childhood SSNS can be attributed to immunosuppressant therapy. Long-term effects on endothelium and renal function are likely understudied. Long-term studies of childhood onset SSNS patients, perhaps by patient registries, are needed to understand the true risk of the disease to adult health.

## Author Contributions

All authors listed have made substantial, direct, and intellectual contribution to the work and approved it for publication.

## Conflict of Interest Statement

KR is site-PI of a Questcor supported research study of patients with nephrotic syndrome. She receives no direct reimbursement from Questcor. The remaining coauthors declare that the research was conducted in the absence of any commercial or financial relationships that could be construed as a potential conflict of interest.
